# Online stimulation of the prefrontal cortex during practice increases motor variability and modulates later cognitive transfer: a randomized, double-blinded and sham-controlled tDCS study

**DOI:** 10.1038/s41598-024-70857-x

**Published:** 2024-08-29

**Authors:** Nisha Maria Prabhu, Nico Lehmann, Elisabeth Kaminski, Notger Müller, Marco Taubert

**Affiliations:** 1 Faculty of Human Sciences, Department of Sport Science, Institute III, Otto von Guericke University, Zschokkestraße 32, 39104 Magdeburg, Germany; 2 Department of Neurology, Max Planck Institute for Human Cognitive and Brain Sciences, Stephanstraße 1a, 04103 Leipzig, Germany; 3 Center for Behavioral and Brain Science (CBBS), Otto von Guericke University, Universitätsplatz 2, 39106 Magdeburg, Germany; 4 Research Group Degenerative and Chronic Diseases, Movement, Faculty of Health Sciences Brandenburg, University of Potsdam, Am Mühlenberg 9, 14476 Potsdam, Germany; 5 Department of Movement Neuroscience, Faculty of Sport Science, Leipzig University, Leipzig, Germany; 6 Neuroprotection Lab, German Center for Neurodegenerative Diseases (DZNE), Magdeburg, Germany

**Keywords:** Cognitive neuroscience, Learning and memory, Sensorimotor processing, Ageing, Human behaviour, Brain, Transcranial magnetic stimulation, Rehabilitation, Randomized controlled trials

## Abstract

The benefits of learning a motor skill extend to improved task-specific cognitive abilities. The mechanistic underpinnings of this motor-cognition relationship potentially rely on overlapping neural resources involved in both processes, an assumption lacking causal evidence. We hypothesize that interfering with prefrontal networks would inhibit concurrent motor skill performance, long-term learning and associated cognitive functions dependent on similar networks (transfer). We conducted a randomised, double-blinded, sham-controlled brain stimulation study using transcranial direct current stimulation (tDCS) in young adults spanning over three weeks to assess the role of the prefrontal regions in learning a complex balance task and long-term cognitive performance. Balance training combined with active tDCS led to higher performance variability in the trained task as compared to the sham group, impacting the process of learning a complex task without affecting the learning rate. Furthermore, active tDCS also positively influenced performance in untrained motor and cognitive tasks. The findings of this study help ascertaining the networks directly involved in learning a complex motor task and its implications on cognitive function. Hence, opening up the possibility of harnessing the observed frontal networks involved in resource mobilization in instances of aging, brain lesion/injury or dysfunction.

## Introduction

Physical activity has proven instrumental in enhancing overall health and well-being across the lifespan. Physical inactivity on the other hand, particularly in the context of aging, has dire consequences extending to cognitive dysfunction^1,2^. Gaining a better understanding of the effects of physical activity on cognitive performance is crucial to support healthy aging, or ameliorate cognitive impairments by incorporating spared mobility into therapy. Perhaps a key component in explaining the link between physical activity and cognition lies within the brain and the synergistic neural networks subserving both motor processing and cognitive functions. Although colocalized brain activity has been identified for motor and cognitive processes, we lack important causal evidence linking movement and cognition at the neural level^3,4^.

Motor learning enables individuals to skilfully perform complex whole-body movements after repetitive practice. Among wide-ranging forms of physical exercise, the influence of complex motor skill learning on cognition has garnered considerable attention. Skill learning has the potential to positively impact cognition through the involvement of key cognitive functions supporting learning, viz., by challenging functions like information processing, decision-making, movement selection, planning, exploring-tracking-switching between courses of actions and predicting outcomes based on experience^5^. While the prefrontal cortex (PFC) has been associated with majority of the above-mentioned cognitive functions^6–9^, this region is also capable of undergoing motor learning-induced brain plasticity. For example, learning a complex and challenging whole-body task (DBT- dynamic balance task) was shown to induce structural and functional changes in the PFC, with PFC structure predicting improved balance task learning^10–12^. Moreover, various training studies suggest transfer effects of motor balance training to relevant cognitive domains^13–15^. The neural overlap hypothesis predicts that behavioural transfer from motor practice to cognitive performance is sub-served by overlapping neural circuits^16–18^; and its underlying mechanisms are hypothesized to occur during the acquisition period of a new skill^19^. Despite these observational neuroimaging and behavioural findings, the causal role of the PFC in motor balance learning and its potential to mediate learning-induced cognitive transfer remains unclear.

Unravelling these complex brain–behaviour relationships have been effectively achieved through non-invasive brain stimulation techniques (NIBS). Transcranial direct current stimulation (tDCS) is one stimulation technique widely employed in the context of motor learning. tDCS involves modulating cortical excitability of a target brain region^20^ in turn affecting behaviour. Anodal tDCS over the primary motor cortex (M1) was shown to enhance motor learning^21,22^. Improved overnight motor skill consolidation has been observed through an effect on networks involved in early consolidation after anodal tDCS over M1^23^. When learning is driven by factors such as performance feedback, sensory feedback and cognitive strategies, variation within the learning process is indicative of an attempt to explore the solution space^24–26^. In such cases, tDCS over PFC has resulted in faster motor learning through regulation of performance variability^27^. tDCS thereby aids in deriving causal inferences in the face of correlative electrophysiological or neuroimaging evidence^28,29^. Although the neurophysiological effects of a single tDCS session are shown to last a few minutes to a couple of hours after the end of stimulation^30,31^, it is nevertheless capable of inducing long-term structural plasticity in the form of rearranged synaptic networks and spinogenesis as established through animal models^32,33^. Similarly, in older adults, training combined with tDCS spread over multiple days was shown to modulate functional connectivity and microstructural brain alterations associated with cognitive performance gains^34^. In line with these findings, prefrontal tDCS applied during balance practice may therefore influence learning-induced prefrontal neural changes that support ongoing balance performance; also affecting transfer to cognitive tasks assessed after a time delay that outlasts the acute neurophysiological effects of tDCS.

In order to test this prediction, cathodal tDCS (c-tDCS) over right PFC (rPFC)^10^ was used during DBT practice sessions in accordance with findings from Kaminski et al.^35^. First, we hypothesized cathodal compared to sham-tDCS (s-tDCS) will affect performance indices and inhibit long-term DBT learning. We have previously shown prefrontal regions to undergo structural changes throughout long-term DBT practice^11^. Therefore, we aim to assess the role of PFC during the process of skill learning using concurrent tDCS over several training sessions. Following the neural overlap hypothesis, we further predict prefrontal tDCS during DBT practice to cause decrements in remote (24 h after motor practice) performance in cognitive tasks that rely on overlapping prefrontal networks. We hypothesize that cognitive domains particularly reliant on prefrontal regions (such as attention^36^, information processing^37^, interference resolution^38^ and cognitive flexibility^39^) are more likely to be affected by DBT learning with concurrent c-tDCS (near cognitive transfer) than domains that are less reliant on processing within PFC. Nevertheless, the latter cognitive domains have also previously been shown to be affected by balance interventions (far cognitive transfer domains like spatial cognition, memory)^13–15^. Likewise, we hypothesize more pronounced motor transfer effects as a result of DBT learning with concurrent s-tDCS as compared to DBT learning during c-tDCS.

**Fig. 1:**
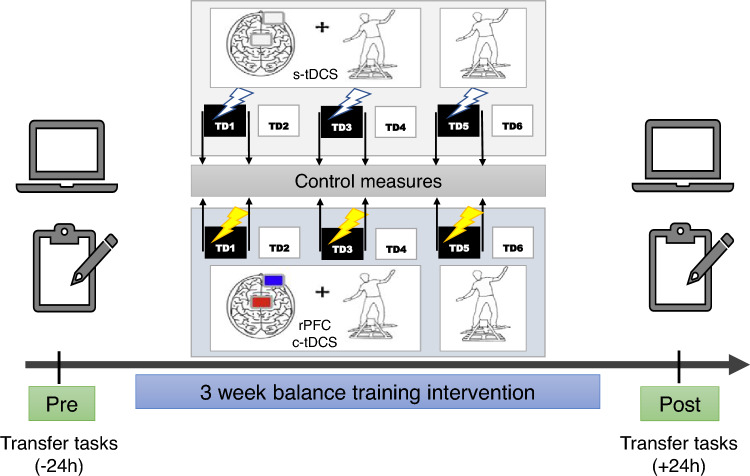
Experimental design: Participants trained on the DBT over 3 weeks with two practice sessions per week. The first session of the week included practice under tDCS stimulation followed 24 h by practice without tDCS. Every session included 15 trials lasting 30 s each, interspersed with a rest period of 90 s. All participants also performed a battery of motor transfer, computer-based and paper–pencil cognitive tests before and after the 6 training sessions.

We extend the paradigm from Kaminski, E et al.^35^, by using a randomised, double-blinded, sham-controlled study to examine the modulatory effect of c-tDCS over the PFC during balance performance and learning over 3 weeks. Additionally, motor and cognitive transfer effects were inspected 24 h pre- and post- the entire training period (Fig. 1).

## Results

Baseline characteristics of the participants in this study with respect to age, gender, body height, body weight, hand dominance and day-to-day physical activities did not differ between groups (Table 1). All the findings from this study related to DBT performance and transfer are summarised in Table 2.

**Table 1:** Demographic data: comparisons between groups in relation to age, gender, height, weight, dominance, physical activity.

Characteristics	Overall	c-tDCS (n = 22)	s-tDCS (n = 22)	c-tDCS vs s-tDCS
Age	21 (3)	21 (2.75)	21 (2.75)	*t*(41.83) = 0.41, *p* = .68
Sex (F; M)	27;17	13;9	14;8	*Χ*^*2*^(1) = 0.09, *p* = .76
Height	174 (15)	173 (16.5)	174.5 (12)	*t*(39.87) = −0.33, *p* = .74
Weight	69 (12.75)	68 (16)	70.5 (10.75)	*t*(40.68) = −0.85, *p* = .39
Hand dominance (left; right)	2;42	0;22	2;20	*Χ*^*2*^(1) = 2.09, *p* = .15
Physical activity: work index	2.19 (1.63)	2.13 (1.31)	2.25 (1.38)	*t*(40.13) = −0.29, *p* = .77
Physical activity: sport index	3.25 (0.88)	3.13 (1.00)	3.25 (1.00)	*t*(41.90) = −0.78, *p* = .44
Physical activity: leisure time index	3.5 (0.75)	3.5 (0.69)	3.75 (0.63)	*t*(40.09) = −0.76, *p* = .45

Values displayed denote the median and interquartile ranges within parentheses for both groups. All statistical comparisons are performed with Brunner-Munzel test except gender and hand dominance (chi-square Χ^2^).

**Table 2:** Dynamic balance task (DBT) and transfer performance results.

Variables	c-tDCS (n = 22)	s-tDCS (n = 22)	c-tDCS vs s-tDCS
Co-efficient of variation (COV)	0.23 (0.06)	0.21 (0.04)	↑ in c-tDCS (*p* = 0.03*, medium effect)
Wii: basic level	120.9 (78.2)	113.5 (69.1)	No difference (*p* = 0.9)
Wii: advanced level	85 (76.6)	61.7 (30.85)	No difference (*p* = 0.4)
CoV vs Wii advanced level	*r* = 0.56, *p* = 0.007*	*r* = −0.16, *p* = 0.46	**Positive correlation in c-tDCS group (medium effect)**
Visual and verbal memory test (VVM): rate of forgetting	0 (11.89)	0 (18.37)	No difference (*p* = .58)
Delta trail making test (ΔTMT)	−1.46 (12.53)	0.03 (16.43)	↓ in c-tDCS (*p* = 0.04*, medium effect)
Trail making test A	−4.79 (5.8)	−4.51 (7.35)	No difference (*p* = 0.52)
Trail making test B	−7.65 (10.75)	−3.85 (12.19)	No difference (*p* = 0.07)
D2- test of attention: concentration score	33.5 (14.5)	31.5 (14.5)	No difference (*p* = 0.32)
Eriksen Flanker task: accuracy interference	0 (0.08)	−0.04 (0.08)	No difference (*p* = 0.06)
Eriksen Flanker task: reaction time interference	−1.78 (27.67)	2.85 (16.05)	No difference (*p* = 0.76)

All statistical comparisons are performed with Brunner-Munzel test or Spearmans rank based correlation.

Significant values are in bold.

### Control measures

#### Stimulation questionnaire

In the questionnaire related to tDCS-induced immediate effects, participants rated on a scale from 0 to 4 their subjective perception of pain, attention, burning sensation, etc. No significant main or interaction effects were found on factors like tingling (*F*(2, 22.42) = 0.17, *p* = 0.84), burning sensation (*F*(2, 19.66) = 0.15, *p* = 0.85), headache (*F*(2, 22.63) = 1.18, *p* = 0.32), concentration problems (*F*(2, 21.15) = 0.58, *p* = 0.57), attention (*F*(2, 17.33) = 0.23, *p* = 0.79), etc. (refer supplementary material 2.1. online for further details).

#### Blinding of stimulation

The blinding index (BI) on training session 1 (TD1) was estimated at 0.56 with 95% CI [0.42, 0.69], on TD3 BI = 0.44 with 95% CI [0.32, 0.57] and on TD5 BI = 0.59 with 95% CI [0.49, 0.69], indicating random guessing (Supplementary Fig. S1 online). These results combined with the results of the stimulation questionnaire, indicate successful blinding between the groups.

#### Stroop task

Controlling for the acute effects of tDCS on general cognitive abilities of the participants, Stroop test was performed as control task immediately before and after tDCS application. Non-significant effect of group, *F*(1, 25.31) = 0.33, *p* = 0.57, time, *F*(2, 22.44) = 0.06, *p* = 0.95, and interaction effect, *F*(2, 22.44) = 1.90, *p* = 0.17, were detected for Stroop accuracy interference reduction. A significant main effect of group was found only for reaction time during the Stroop task, *F*(1, 25.32) = 7.53, *p* = 0.01, without an effect of time, *F*(2, 22.77) = 0.35, *p* = 0.71, or interaction, *F*(2, 22.77) = 0.14, *p* = 0.87 (Supplementary Fig. S2 online). This result stems from the poor performance of the s-tDCS group immediately after training compared to pre-training performance (c-tDCS group performance remained unchanged). This pattern remained consistent over time.

#### BESS task

Balance Error Scoring System (BESS) was performed as a control task immediately before and after tDCS application to control for acute tDCS effects on general balance ability. tDCS stimulation did not affect general balance ability between groups as no effect for factor group (*F*(1, 25.96) = 0.71, *p* = 0.40), time (*F*(2, 22.04) = 1.18, *p* = 0.33) nor an interaction effect (*F*(2, 22.04) = 1.58, *p* = 0.23) was observed (Supplementary Fig. S3 online).

### tDCS effects on DBT performance

#### DBT learning

**Fig. 2:**
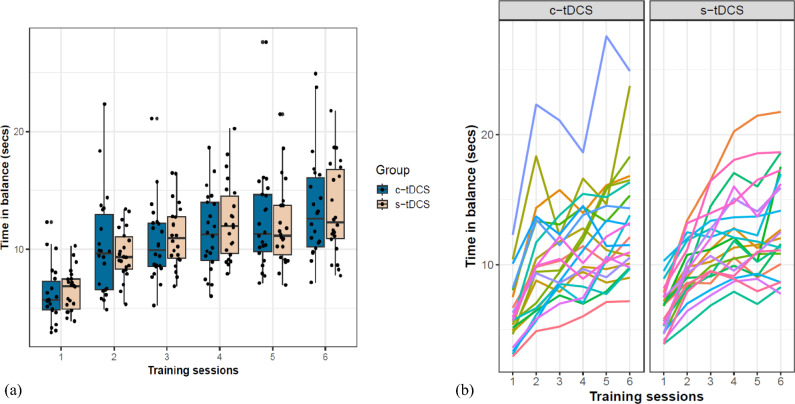
DBT performance and learning: (**a**) improvements in Time in Balance (TIB) from training day-1 to training day-6. Every data point represents mean TIB values for each participant at every training session, (**b**) trajectory of performance improvements for every participant over six training sessions in the c-tDCS and s-tDCS groups, respectively. Each line represents the performance trajectory of a different participant. Training sessions 1, 3, 5 on the x-axis correspond to DBT training sessions with concurrent tDCS (TD1, TD3, TD5).

Baseline performance recorded as the first two trials on TD1 (before tDCS commenced) was found to be similar between both groups (mean time in balance (TIB) c-tDCS: 3.05 ± 1.7 secs vs s-tDCS: 2.99 ± 1.49 s), Brunner-Munzel *t*(41.97) = −0.27, *p* = 0.78, δ = 0.05 (Supplementary Fig. S4a online). After six consecutive training sessions on the stabilometer, both groups significantly improved their DBT performance, *F*(5,18.66) = 34.57, *p* = 0.00, *d* > 1.81 (mean TIB c-tDCS: 13.53 ± 4.5 secs and s-tDCS: 13.36 ± 3.93), without an effect of group (*F*(1,25.55) = 0.13, *p* = 0.73, *d* = 0.11) or group*time interaction effects, *F*(5,18.66) = 1.29, *p* = 0.31, *d* = 0.35 (Fig. 2a).

#### DBT performance variability

**Fig. 3:**
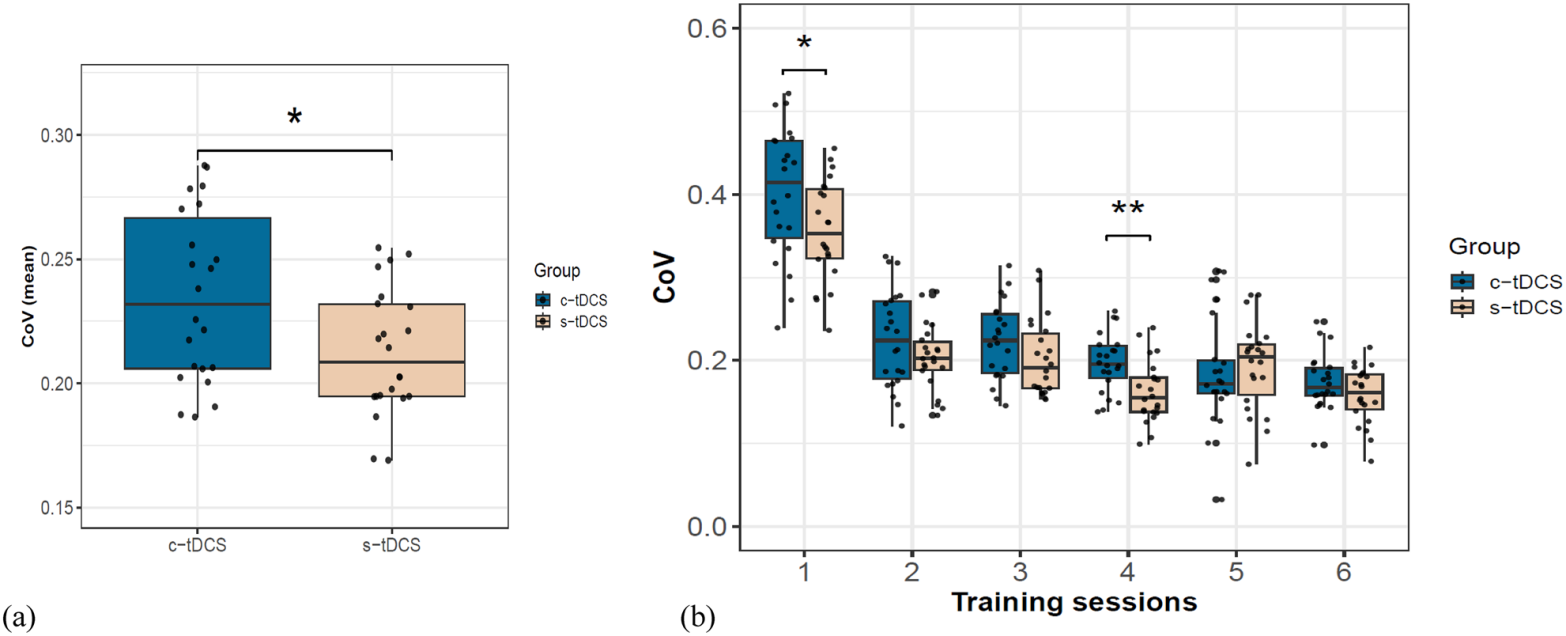
Performance variability expressed as coefficient of variation (CoV = SD/mean): (**a**) Mean CoV over the entire training duration shows significantly higher variability exhibited by the c-tDCS group, Brunner-Munzel *t*(38.16) = −2.22, *p* = 0.03, δ = 0.36 (medium), (**b**) reduction of CoV over the 6 training sessions. Asterisks indicate significant differences in variability between both groups at that specific training session (* ≤ 0.05, ** ≤ 0.001). Training sessions 1, 3, 5 on the x-axis correspond to DBT training sessions with concurrent tDCS (TD1, TD3, TD5).

Due to the apparent dissimilar improvement trajectories observed between both groups (Fig. 2b), the displayed performance variability for each participant was quantified using coefficient of variation (CoV). The baseline CoV calculated from the first two trials on TD1 (before tDCS commenced) was similar between both groups (mean CoV c-tDCS: 0.30 ± 0.2 vs s-tDCS: 0.27 ± 0.14), Brunner-Munzel *t*(34.02) = -0.29, *p* = 0.77, δ = 0.05 (Supplementary Fig. S4b online). However, over the entire training duration, the c-tDCS group (0.24 ± 0.03) on average exhibited significantly higher performance variability compared to s-tDCS group (0.21 ± 0.025), Brunner Munzel *t*(38.16) = −2.22, *p* = 0.03, δ = 0.36 (medium) (Fig. 3a). Across the six training sessions (Fig. 3b), the c-tDCS group displayed higher CoV than the s-tDCS group, *F*(1,22.79) = 4.91, *p* = 0.04, *d* = 0.68. CoV reduced significantly over time for both groups *F*(5,19.36) = 52.07, *p* < 0.001, *d* = 2.23, revealing a group*time interaction, *F*(5,19.36) = 2.96, *p* = 0.04, *d* = 0.53.

Fisher’s chi-square combination of rank-based partial *p*-values^40–42^ across training sessions yielded a significant effect for group difference in performance variability (*p* = 0.01). Posthoc unadjusted and multiple testing adjusted comparisons revealed significant differences at TD1 (Brunner-Munzel *t*(34.20) = 2.16, *p* = 0.02, δ = 0.4 (medium)/*p*FWE = 0.09) and TD4 (Brunner-Munzel *t*(39.94) = 3.45, *p* = 0.001, δ = 0.5 (large)/*p*FWE = 0.03) displaying higher variation in the c-tDCS group performance than the s-tDCS group (Fig. 3b). No significant group differences were found at TD2 (Brunner-Munzel *t*(34.76) = 0.95, *p* = 0.16/*p*FWE = 0.40), TD3 (Brunner-Munzel *t*(41.99) = 1.66, *p* = 0.04/*p*FWE = 0.17), TD5 (Brunner-Munzel *t*(41.69) = −0.85, *p* = 0.80/*p*FWE = 0.81) and TD6 (Brunner-Munzel *t*(41.66) = 1.21, *p* = 0.12/*p*FWE = 0.3).

Further analyses show that initial performance did not influence the observed between group differences after DBT training with/-out c-tDCS interventions (Refer supplementary materials 2.6 online). Moreover, after controlling for baseline motor skills (pre-intervention BESS scores), the effect of tDCS on performance variability remained significant, *b* = 0.022, *t* = 2.14, *p* = 0.04 (Refer supplementary materials 2.7 online).

### Effect of concurrent tDCS on cognitive transfer

#### Visual and verbal memory test (VVM)

Baseline performances were similar for both groups in delayed recall (Brunner-Munzel *t*(35.14) = 0.61, *p* = 0.54, δ = 0.11) and rate of forgetting (Brunner-Munzel *t*(40.82) = 0.22, *p* = 0.82, δ = 0.03). The 3-week intervention did not lead to any groups differences with respect to either delayed recall, Brunner-Munzel *t*(37.64) = −1.44, *p* = 0.16, δ = 0.25, or the rate of forgetting, Brunner-Munzel *t*(39.21) = 0.56, *p* = 0.58, δ = 0.1 (Supplementary materials 2.10.1 online, Supplementary Fig. S8 online).

#### Trail making test (TMT)

##### ΔTMT

**Fig. 4:**
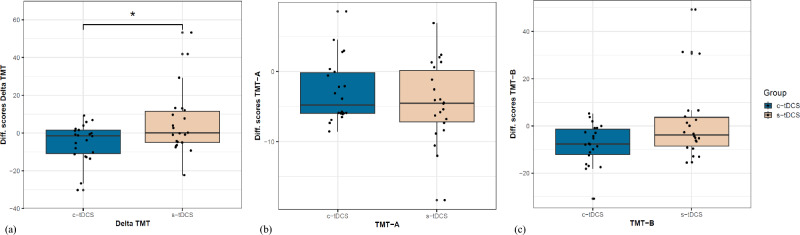
Effect of the interventions on Trail-making test A & B performances: (**a**) improvement in Delta TMT (Δ TMT = TMT-B – TMT-A) seen as pre-post difference scores calculated from the pre and post test scores expressed in seconds. Lower scores signify higher improvements. Asterisks indicate significant difference between groups. (**b**) Similar pre-post TMT-A difference scores for both groups; (**c**) pre-post TMT-B difference scores for both groups displaying a tendency towards higher improvements in c-tDCS as compared to s-tDCS. Here lower scores signify good performance.

No between group differences were detected in baseline performances in ΔTMT (Brunner-Munzel *t*(36.57) = 0.98, *p* = 0.33, δ = 0.18). However, a statistically significant post-intervention improvement in ΔTMT was detected in the c-tDCS group compared to the s-tDCS group (Fig. 4a), Brunner-Munzel *t*(40.49) = 2.08, *p* = 0.04, δ = 0.34 (medium).

##### TMT-A

No significant differences between groups were observed at either baseline (Brunner-Munzel *t*(41.78) = 0.48, *p* = 0.63, δ = 0.08) or post-intervention in this subtest, Brunner-Munzel *t*(39.89) = −0.64, *p* = 0.52, δ = 0.12 (Fig. 4b).

##### TMT-B

In this subtest measuring cognitive flexibility, faster completion times were exhibited by the c-tDCS group compared to s-tDCS group although this difference did not reach statistical significance (Fig. 4c), Brunner-Munzel *t*(41.49) = 1.89, *p* = 0.07, δ = 0.31 (medium). Since the baseline performance in TMT-B was similar for both groups (Brunner-Munzel *t*(38.38) = −0.64, *p* = 0.53), justifications for such asymmetric performance improvement other than training under concurrent tDCS seem unlikely.

#### D2-test of attention (d2)

**Fig. 5:**
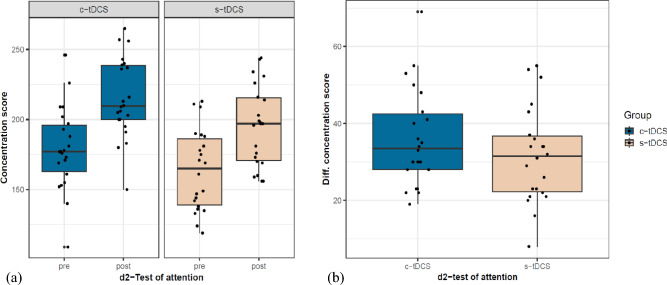
Concentration score measured using d2-test of attention was considered as a parameter of attention; (**a**) pre- and post-test concentration scores for both groups, (**b**) improvement in concentration scores seen as pre-post difference scores for both groups.

No between-group differences were detected at baseline in the concentration scores (Brunner-Munzel *t*(39.14) = 1.68, *p* = 0.1, δ = 0.3). Both groups equally improved post-intervention (Fig. 5a), as no between-group differences were observed in the concentration scores, Brunner-Munzel *t*(40.67) = −0.99, *p* = 0.32, δ = 0.17 (Fig. 5b).

#### Eriksen Flanker task

No between group differences were observed at baseline in this task (Accuracy interference: Brunner-Munzel *t*(40.14) = 0.29, *p* = 0.78, δ = 0.05; reaction time interference: Brunner-Munzel *t*(40.51) = 1.01, *p* = 0.32, δ = 0.18).

**Fig. 6:**
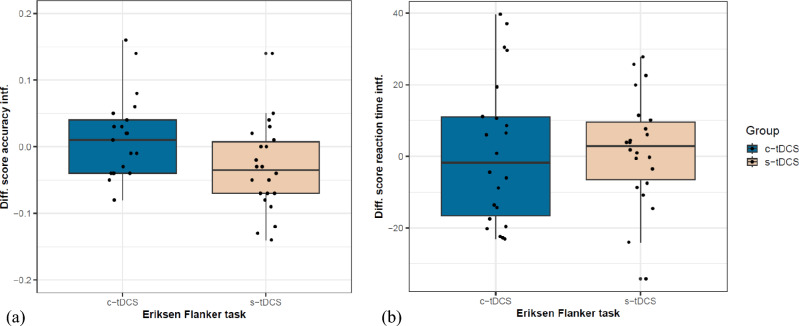
Accuracy interference and reaction time interference scores were considered as parameters of interest in the Eriksen flanker test: (**a**) Improvement in accuracy interference scores seen as pre-post difference scores calculated for both groups. For purposes of better visualization an outlier (−0.68) from the c-tDCS group was removed from the graph, (**b**) improvement in reaction time interference scores seen as pre-post difference scores.

Accuracy interference. c-tDCS group showed comparatively lower improvements than the s-tDCS group in accuracy interference reduction after the intervention trending towards significance (Fig. 6a), Brunner-Munzel *t*(41.83) = −1.93, *p* = 0.06, δ = 0.32 (medium). Additionally, a significant positive correlation was observed between the accuracy interference score and the mean CoV only in the s-tDCS group, p = 0.02, r = 0.48 (Supplementary materials 2.10.2 online, Supplementary Fig. S9 online).

Reaction time interference. No difference between either groups was observed for this reaction time metric of the Eriksen flanker task, Brunner-Munzel *t*(35.40) = 0.31, *p* = 0.76 (Fig. 6b).

### Effect of intervention on motor transfer

**Fig. 7:**
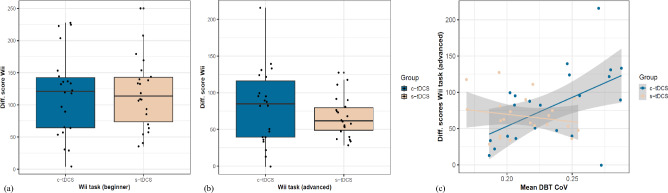
Effects of the interventions on performance of the football header task (Nintendo Wii): the Wii score is a cumulation of all the hits of the target objects and unsuccessfully dodged non-target objects; (**a**) Improvement in Wii scores at the beginner level, (**b**) improvement in Wii scores at the advanced level are expressed as the difference in the pre to post test for both groups. (**c**) Correlation between performance variability (CoV) on the stabilometer and Wii change scores (advanced level; pre to post intervention) for the c-tDCS and s-tDCS groups.

Wii task. No between-group differences were observed at baseline in this task (beginners: Brunner-Munzel *t*(35.18) = 1.33, *p* = 0.19, δ = 0.24; advanced: Brunner-Munzel *t*(40.97) = 0.47, *p* = 0.64, δ = 0.08). At the beginners level, both groups equally profited from the intervention (c-tDCS median = 120.9 ± 78.2 points vs s-tDCS median = 113.5 ± 69.1 points), Brunner-Munzel *t*(40.34) = 0.15, *p* = 0.9, δ = 0.03 (Fig. 7a). Similarly at the advanced level, no between-group differences in improvements were observed post intervention (c-tDCS median = 85 ± 76.6 points vs s-tDCS median = 61.7 ± 30.85 points), Brunner-Munzel *t*(28.75) = −0.79, *p* = 0.4, δ = 0.15 (Fig. 7b).

However, the difference scores at the advanced level revealed a clear distinction with-in the c-tDCS group where 13 participants exhibited larger improvements (≥ 80 points) compared to 9 participants with minimal gains (≤ 50 points). This distinction was absent in the s-tDCS group. A subsequent Spearman rank-based correlational analysis revealed a positive medium correlation between the difference scores of the Wii task (advanced level) and mean performance variability (CoV) over the entire training session on the stabilometer, which was only present in the c-tDCS group, *r* = 0.56, *p* = 0.007, not observed in the s-tDCS group (*r* = −0.16, *p* = 0.46). This suggests that participants from the c-tDCS group exhibiting larger variability during stabilometer practice also displayed higher gains in the Wii task (Fig. 7c).

## Discussion

This randomised, double-blinded, sham-controlled tDCS study highlights the importance of frontal networks in learning a complex dynamic balance task. Our results demonstrate that the influence of c-tDCS over these networks during the process of long-term motor learning caused higher performance variability compared to the s-tDCS group. This increase in behavioural variance indicates that the stimulation causally affected (pre-)frontal brain networks^27,28^. Moreover, DBT training with concurrent c-tDCS not only resulted in motor transfer effect on postural control, but also led to near cognitive transfer on the cognitive flexibility domain known to rely on the prefrontal networks. These transfer effects persisted 24 h after the end of training.

In this study, tDCS applied during DBT practice was aimed at influencing networks implicated in long-term DBT learning. These networks were selected based on previous findings that show macro- and microstructural properties of PFC-SMA regions predict future DBT learning^10,43^ also changing in response to DBT practice^11,44,45^. By administering tDCS during task execution, we tried to effectively target the task-relevant networks involved during learning; in turn modulating learning-induced synaptic plasticity in these networks^28,46^. Although previous studies demonstrated through mediation analyses^47^, evidence of a brain-behaviour relationship between PFC-SMA networks and balance learning, the neuroimaging findings remain correlative. However, a single session of online c-tDCS over the right PFC-SMA region during training had an acute effect on subsequent DBT performance^35^. Here, we extend these previous findings by causally showing PFC-SMA network involvement in long-term balance learning, manifested through increased performance variability^28^.

Due to dissimilar amplification in neuronal noise, the true direction of the effect of tDCS on performance may be varied across and with-in participants. In such cases, the sheer increase in variance (beyond measurement noise) after tDCS may be considered as evidence for a cause–effect relationship^28^. Such behavioural consequences of tDCS may arise due to individual differences in the recruitment of brain networks during task performance, leading to differences in excitability modulation^20,28,48^. Along with reported within-session, non-linear effects of c-tDCS^49^, dissimilarities in tDCS-induced modulation of cortical excitability may not necessarily translate into behavioural deviations as drastic as performance inhibition. Lack of DBT performance deterioration in this study can therefore be associated with tDCS being a weak direct current and its behavioural effects meagre. This may allow networks to capably compensate for weak disturbances during online stimulation by adapting to the electric field over time^28^. The results of this study however demonstrate improved DBT performance for both groups over the 3-week training duration; indicating similar task proficiency at the end of practice. Hence, tDCS may have affected the process of learning a complex task rather than changing the learning trajectory.

The prefrontal networks that support the strategy building aspect of motor learning were the prime targets of c-tDCS in our study^24,46^. Consequently, participants were not instructed on the most optimal task execution strategy (contrary to a ‘classical’ motor skill learning/training), instead, encouraged to learn the task by discovering their own strategies via trial and error^50^. Previous studies investigating the mechanisms involved in adopting specific courses of action during learning have associated the anterior PFC in exploration of new possibilities. Here, future outcomes are said to be predicted by tracking alternative options and exploratory switching between courses of actions through extrapolation of short-term trends^7,9^. Hence, task complexity and uncertainty of outcomes may dictate the extent of PFC involvement, where selection of appropriate strategies and guiding cognitive resources to implement these strategies is done by integrating and comparing various sequential outcomes^6,9^. Owing to the task complexity and the available solution space, the DBT fulfils criteria’s particularly conducive for cognitive processes involved in reinforcement learning, which in our case involves exploration of solutions achieved through various coordinative whole-body movements. Therefore, we speculate that PFC-dependent networks responsible for exploration of new performance strategies (in the context of learning) were modulated by c-tDCS. This modulation was behaviourally expressed as increased performance variability.

Furthermore, it is suggested that extending learning gains to untrained tasks is only possible if a shared commonality exists between these tasks, viz., abilities required in executing both tasks, neural processing mechanisms and brain regions^16,17,51^. These transfer effects are also theorised to be tied to early phases of structural plasticity within overlapping networks^19^. The ‘neural overlap hypothesis’ has previously been supported by evidence from studies applying concurrent tDCS during cognitive training which resulted in microstructural brain alterations alongside near-transfer behavioural effects^34,52^. Since the motor learning paradigm used in this study is capable of inducing structural grey and white matter changes in PFC and SMA regions^11,45,53,54^, we further hypothesized it to potentially lead to cognitive transfer effects in tasks relying on the affected regions. Consistent with this hypothesis, we found higher improvement in executive functioning performance (i.e., ΔTMT)^55^ as a result of DBT training with concurrent rPFC c-tDCS compared to s-tDCS. Similarly, both aerobic exercise on its own^56^ and a-tDCS over left DLPFC during coordinative exercise^57^ have previously shown a tendency towards TMT performance improvements. Likewise, cognitive training combined with tDCS at an intensity of 1.0-mA augmented both decision-making performance and cognitive transfer^58^.

Despite a global network involvement in TMT execution^59^, our regions of interest were restricted to the overlapping PFC-SMA networks involved in DBT learning. We hypothesize the combination of DBT training-induced plasticity, discovery-learning based motor training and tDCS to encourage a rapid network reorganisation and compensation^60–62^. This functional compensation probably constituted conditioning new or otherwise inactive networks within the overlapping brain regions leading to an advantageous effect of intervention, absent in the s-tDCS group^63,64^. Richly connected brain networks supporting a multitude of cognitive functions required in TMT execution may have created a potential for transfer via compensatory mechanisms in the overlapping networks^59,65–67^. A combination of brain imaging and stimulation techniques is nevertheless essential to prove the involvement of specific functional and structural correlates of PFC in learning and associated transfer.

Contrary to the observed effect on executive functioning, we did not find significant between-group differences on memory or attention abilities, although positive effects of physical exercise (e.g., coordinative and aerobic exercise) on visuospatial attention, working memory^68^, associative memory, spatial cognition^14,15^ and visuospatial memory^69^ have been observed in previous studies.

Finally, the observed transfer effects on PFC-SMA-dependent cognitive tasks can be assumed to be due to a shared commonality with the trained task (neural overlap hypothesis)^19,51^, which changed as a function of the intervention, demonstrating a potential common neural substrate underlying the trained balance task and the transfer task^70^. This complex motor training engaging higher-order processes may have enabled cognitive improvements by transferring learning gains to untrained tasks. In turn benefiting abilities like information processing, goal-dependent inhibition/ maintenance of responses, formulating strategies based on feedback, distributing attention over multiple strategies, switching between strategies (cognitive flexibility), etc.^16,17,51^. Similar findings from^14^ demonstrate balance training-induced improvement in memory and spatial cognition attributed to a training that encompassed proprioceptive, visual and motor-based learning. Likewise, a month of slackline training improved vestibular-dependent spatial orientation performance^13^ suggesting a positive effect on vestibulo- hippocampal spatial orientation.

Lastly, we also observed a motor transfer effect on an untrained balance task (Nintendo Wii header game- advanced level) in the c-tDCS group compared to the s-tDCS group. Interestingly, consistent with the ‘neural overlap hypothesis’, in the c-tDCS but not in the s-tDCS group we observed a medium-sized positive correlation between DBT performance variability and Wii scores. Such motor transfer effects have recently been observed by^71^, manifested as improved cross-limb transfer from the trained to the untrained hand after anodal tDCS over rM1. Similarly, we hypothesize that participants in our study exhibiting higher DBT performance variability were able to successfully use the movement solutions learned during DBT training onto an untrained balance task which also requires a comparable movement pattern in terms of body’s centre of mass (COM) control and displacement.^72–74^ highlight the introduction of variation during practice as a key aspect in eliciting new movement solutions enabling a degree of transfer beyond the practiced solutions. However, further studies are required to support the role of movement variability to improve transfer during stabilometer learning.

Although the results of this study highlight the importance of the frontal networks in learning a complex task, we are unable to disentangle the contributions of PFC from those of SMA as both these regions have been implicated with undergoing learning-induced structural changes^11^. Even though our near cognitive transfer results do point towards higher PFC involvement, we were not able to definitively outline the specific contributions of these regions. Further work utilizing a combination of tDCS and neuroimaging may aid in explicitly mapping stimulation-induced changes at the neuronal and network levels. Linking these brain changes to the behavioural effects would be the natural subsequent step in order to unravel the complexity of the underlying brain-behaviour relationship. Stimulating an alternative brain region is usually advised in order to ascertain that the observed effects emanate solely as a result of interference within the regions of interest^28,29^. However, this control condition was not included since we intended on influencing the networks previously implicated in learning the complex DBT. In addition, among various brain regions reported to undergo learning-dependent plastic changes^11^, c-tDCS over PFC was shown to cause decrements in DBT learning^35^. However, neither a-tDCS nor s-tDCS over PFC led to interference in DBT learning, with no significant differences between these conditions. Therefore, only c-tDCS over PFC in combination with s-tDCS as the active control group was included in our paradigm. Nevertheless, we acknowledge that the inclusion of an active control group would have further strengthened the interpretation of our findings. In light of the recently revealed predispositions to improved learning abilities^10,43^, heterogeneity of participants in the form of genetic makeup, brain structure and environmental diversity requires consideration^75^. Moreover, the solitary effect of tDCS on cognitive abilities without the influence of training is an aspect that could help differentiate between the cumulative effect of tDCS and training observed in this study.

Our results provide new evidence for PFC-SMA involvement during long-term DBT practice. Specifically, we show that interfering with these networks using c-tDCS led to increased performance variability, potentially indicating a causal involvement of PFC-SMA networks in DBT learning^28^. Against the background of ‘neural overlap hypothesis’, we interpret the observed tDCS-effects on motor and cognitive performance as effects pertaining not only to the trained tasks, but also to the untrained tasks relying on overlapping brain networks. The conclusions drawn through this study reinforce the positive impact of physical activity on cognition through the synergistic neural networks sub-serving both motor processing and cognitive functioning. An understanding of this brain-behaviour relationship may prove valuable not only in promoting overall health through exercise but also support healthy aging by means of mobilizing neural resources to remedy dysfunction.

## Material and methods

### Ethics statement

This study was approved by the local ethics committee of the Otto-von-Guericke University, Magdeburg [130/20]. The study was retrospectively also registered in the German Clinical trial Register (DRKS-ID: DRKS00033716; date of registration: 26/02/2024). Conforming to the declaration of Helsinki, all subjects provided their written informed consent prior to participation in the experiment and received financial compensation for participation. The entire study was conducted at the lab facilities under the chair of Training science (Cognition and action), Department of Sport Science, Otto von Guericke University, Magdeburg.

### Study design

We conducted a randomised, double-blinded, sham-controlled study to examine the modulatory effect of c-tDCS over the PFC during balance performance and learning over 3 weeks in 44 subjects between the ages of 18–35 years (n = 44, 21.8 ± 3.25years, 27 females). Sample size was estimated based on findings from^35^ using a similar motor learning paradigm along with concurrent tDCS (supplementary materials 1.1. online for further details). Highly skilled subjects such as slackliners or participants with prior experience with the DBT were excluded. Additionally, in order to evaluate their general physical activity levels, participants were required to fill-in an activity questionnaire^76^.

All participants were informed about potential risks of non-invasive brain stimulation used in this study. After granting their written informed consent, participants were randomly assigned to either cathodal (c-tDCS, n = 22) or sham (s-tDCS, n = 22) groups by one of the authors by drawing lots (MT: no contact with any of the participants). Neither the researchers involved in data acquisition/training nor the participants were aware of the group assignment. Irrespective of the training groups, similar tDCS electrode montage using EEG 10–20 position was applied. The entire training duration lasted a total of 3 weeks consisting of two training sessions per week (TD1-TD6) with motor and cognitive transfer tests conducted 24 h pre- and post- the training period (Fig.  1 ). The first training session of the week (TD1, TD3, TD5) included DBT practice with concurrent c-tDCS or s-tDCS over right PFC (rPFC). These training sessions were followed (24 h later) by a re-evaluation of the DBT performance without c-tDCS (TD2, TD4, TD6). To control for the acute effects of tDCS on general balance ability and general cognitive abilities of the participants, balance and cognitive assessments were performed as control tasks immediately before and after tDCS application (refer “Control measures”).

#### Complex balance task (DBT)

The motor learning paradigm in our study included a whole-body dynamic balance task consisting of a balance platform that moves in a see-saw like manner known as a Stabilometer (stability platform, Model 16030, Lafayette Instruments, Lafayette, IN, USA), with a maximum deviation of 26° on each side. A typical training session on the stabilometer included 15 trials lasting 30 s each, with 90 s rest period between each trial. The goal was to maintain the platform in a horizontal position, i.e., parallel to the floor, for as long as possible during the 30 s trial; staying within a target deviation of 0°–3° to the right or left from the horizontal axis. This required the participant to position the body’s centre-of-pressure vertically above the boards’ axis of rotation. Each training session lasted a maximum of 30 min each day. At the end of each trial, participants received feedback about their performance in the form of time in balance (TIB-outcome measure), i.e., seconds spent within the ± 3° target window. Receiving no instructions regarding task performance strategies, apart from the necessary safety guidelines and TIB feedback, they were granted the freedom to explore their own strategies in order to improve performance over the six training sessions (Discovery learning approach)^50,77^.

#### Transcranial direct current stimulation (tDCS)

**Fig. 8:**
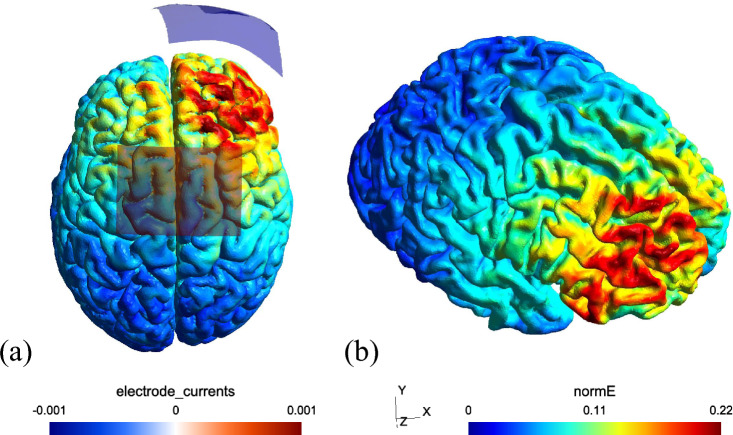
tDCS current flow simulation: Modelled distribution of current projected on a standard head model. (**a**) Cathode is represented via the blue rectangle over the right prefrontal region (Fp2) whereas the anode is depicted using the red rectangle (FCz), (**b**) normalised electric field strength (normE) is indicated through colormaps ranging from lowest (blue) to highest (red) field strengths. The current flow image was created using the SimNIBS software version 3.2^78^.

A weak direct current of 1 mA generated from a rechargeable battery driven stimulator (NeuroConn Gmbh, Ilmenau, Germany) was used for a total duration of 20 min during TD1, TD3, TD5. Electrodes were fastened using Velcro straps over the areas corresponding with rPFC (EEG 10–20 electrode placement), i.e., cathodal electrode on the right supraorbital region (Fp2). The reference electrode was placed midway between frontal and central zero (FCz with slight off-set to left side) ensuring no overlap with the cathodal electrode occurred while simultaneously avoiding stimulation over the M1 area^21^. Electrodes were encased within sponge covers drenched in saline solution (NaCl) and rehydrated intermittently if necessary using syringes without moving the electrodes from their fastened position. Sizes of both electrodes were kept at 35 cm^2^ (5 × 7 cm) with a current density of 0.028 mA/cm^2^ and a total charge of 0.033 C/cm^2^ under each electrode, similar to^35^. The cathodal stimulation group (c-tDCS, n = 22) experienced stimulation with a trapezoidal pulse form consisting of ramp-up at the beginning and ramp-down lasting 30 secs at the end of 20-min stimulation period. However, the s-tDCS group (n = 22) received a similar ramp-like stimulation with a fade-in, maintenance of stimulation for 30 secs only, followed by a fade-out. Figure 8 shows the simulated tDCS electric field distribution based on a finite element model of a representative head ‘Ernie’ inside the open-source SimNIBS software^78^. For the simulation, the cathode was placed on Fp2, the anode was placed on FCz. This model demonstrates that maximum electric field strength (0.2 V/m) was concentrated under the cathode targeting the rPFC. The tDCS stimulation was started only after the second trial during each training session and lasted 20 min thereafter. The participants carried the stimulator in a backpack during DBT practice. As a precautionary measure, a questionnaire pertaining to sensory perception, changes in attention, perception of fatigue and discomfort after/ during stimulation was administered^79^. To assess the success of blinding, all participants were asked whether they believed they received stimulation or not after TD1, TD3 and TD5.

#### Control measures

Acute effects of tDCS stimulation on general balance ability and executive functions were tested using the Balance Error Scoring System (BESS)^80^ and the Stroop test^81,82^ respectively. These tests were administered pre- and post- training sessions where participants received tDCS (refer supplementary materials 1.2 online for test description). These tasks were chosen to match our tasks of interest with respect to its characteristics and difficulty, although distinct in terms of the involved cognitive or motor functions of interest. This allowed us to ascertain task specificity while examining the acute effects of tDCS, in turn avoiding confounds via co-affected supporting functions^28^. Additionally, pre-intervention BESS scores were used as measures of baseline motor/balance skills.

#### Transfer tests

Based on transfer effects reported in previous coordinative exercise training studies with and without tDCS^4,14,18,57^, a cognitive test battery conducted 24 h before and after the training period investigated the transfer effects of concurrent tDCS and motor practice. The tests and the measured parameters included:

- Visual and verbal memory test (VVM^83^): evaluates short-term maintenance of memory consisting of two subtests assessing visuospatial memory and verbal memory separately. Only the visuospatial subtest represented by a street map was used in this study as numerous studies have shown coordinative training^14,68^, aerobic training^69^ as well as tDCS-based^84^ interventions to influence working memory, in particular visuospatial memory functions. This subtest requires the participants to memorise a given path on a street map and recall it (immediate and delayed) on an identical but empty street map. Parallel forms of the subtest (theatre & museum) were employed for pre- and post-testing. Encoding time was fixed at 2 min shortly followed by time for retrieval (free immediate recall-VVM1) timed at 2 min to completion while delayed recall (VVM2) of the material was performed 30 min later. The performance measures of interest were the number of correctly recalled intersections in the street map, with a maximum of 31 correct intersections, and rate of forgetting calculated as a percentage of the immediate and delayed recall using:
  $$ {\text{Rate of forgetting }} = \, \left( {{\text{VVM2 }}{-}{\text{ VVM1}}} \right)/{\text{ VVM1}} \times {1}00 $$
- Trail making test (TMT^85,86^): is a measure of divided attention and scanning abilities, with particular focus on cognitive flexibility involving switching between sets of letters and alphabets. The neural correlates of this task also largely overlap with the areas of interest in this study^59,87,88^. This test was conducted in 2-parts: (i) TMT-A requires the participant to connect randomly scattered numbers from 1 to 25 in an ascending order (1–2–3–…); (ii) TMT-B requires the participant to alternate between letters (A-L) and numbers (1–13), i.e., connecting a number to an alphabet proceeding in an ascending order (for numbers) and alphabetical order, constantly switching between these sets (1-A–2-B–3-C–…). The participant is required to familiarise themselves with shortened versions of each part before they begin. They are instructed to perform the task as fast as possible with minimal errors without lifting the pen off the sheet. Time to completion and difference between the time required to complete parts A & B (delta/Δ TMT) are used as variables of interest.
- D2-test of attention: assesses sustained attention and visual scanning speed and accuracy^89^. Findings from^36^ demonstrated cardiovascular exercise-induced frontotemporal plasticity to mediate improved attention measured using this test, also assumed to be an ability vital in learning the DBT. We used the German paper and pencil version of the revised “d2 Test of Attention” (d2-R^90^) which consists of 14 test lines with 47 symbols per line. The symbols can be either of the lowercase letters “d” or “p” marked with 1, 2, 3, or 4 small dashes above and/or below the letter. The participant is required to strike through occurrences of the letter “d” bearing 2 dashes only as quickly and accurately as possible. All other symbols act as distractors to be ignored. The participants were requested to proceed from left to right with 20 s dedicated to each line; after which they need to proceed to the next line. Concentration performance was the variable of interest, defined as the number of marked distractors (sum of errors of commission and errors of overlooking) deducted from the total number of processed targets.
- Eriksen Flanker task^91^: addresses interference resolution ability, a more selective inhibition process, where task-relevant responses are maintained while task-irrelevant stimuli and goal-irrelevant responses are inhibited^38,92^. Exercise interventions have not only shown to positively impact this interference resolution ability^3,4^, but the neural correlates of this task also correspond with areas previously shown to undergo structural changes after DBT learning^11,38,54^. In this task, the participants were presented with a fixation arrow at the centre of the screen which was then replaced by a stimulus cue (> or <). Here they were asked to respond only to the stimulus cue not the flanking array of arrows on either side. The trials consisted of either flanking arrows pointing in the same direction as the central stimulus cue (congruent trial: <  <  <  < <), or flanking arrows pointing in the opposite direction (incongruent trials: >  >  <  > >). Each participant underwent a familiarisation block of 10 trials followed by two successive blocks with 50 trials each, first-order counterbalanced such that congruent and incongruent trials followed each other equally as often. Keeping the target stimulus as simple as possible with ‘ < ’ representing left button press and ‘ > ’ representing right button press avoided incorrect responses due to failure of learning the correct responses; hence focusing on target identification, information processing followed by the effect of response selection conflict^91^.
- Wii task (motor transfer test): a football header task available on the WiiFit console was used to assess the goal-directed control of COM movement/sway of the participants (accuracy as well as reaction time), similar to movement control required during execution of the DBT. A Wii Nintendo connected to a large TV screen was used for this task. The participants were asked to stand with both feet on the Wii-board where their centre of mass was tracked and represented as an avatar on the screen with the goal of heading balls kicked in their direction in order to gain points. Simultaneously, other objects (panda masks and shoes) were also added to the mix with instructions to dodge these objects or risk losing a point. These objects were tossed either laterally (left or right) or in the centre, prompting the participant to accurately realign the avatar in the required direction by shifting their COM/ body weight on the Wii board. Participants performed 10 trials with 30 s breaks in between. The total score, a measure of both accuracy and reaction time, is derived through the successful hits and misses of objects during each trial.

### Data analysis

All statistical analyses for this study were conducted using the software R version 4.1.3^93^. Due to non-normal distribution of data in our study, robust non-parametric statistical methods were used for all analyses^94,95^. Between group comparisons at baseline for all demographic variables were conducted, depending on the scale level, using chi square or non-parametric Brunner-Munzel^40^ tests. To investigate the performance changes over the entire training duration and on the data from the questionnaire inspecting perceived sensory effects of stimulation, robust two-way mixed ANOVAs based on 20% trimmed means as implemented in the WRS2 package^96^ were used. The blinding responses were analysed using BI package implemented in R. James blinding index (two-sided) for TD1, TD3 and TD5 are reported separately and interpreted as 0.0 = complete unblinding, 0.5 = random guessing and 1.0 = complete blinding^97^.

The TIB recorded during 15 trials was averaged for each TD for between and with-in group comparisons. In addition to TIB, coefficient of variation (CoV = SD/mean) in TIB over each TD was compared. A posthoc analyses of CoV’s at every TD was further conducted using the nonparametric combination (NPC) framework, aggregating results from multiple studentized Wilcoxon permutation tests (Brunner Munzel tests)^40,41^ using Fisher’s chi-square^42^ combination into a single global *p*-value while accounting for the dependence among the component tests (R package NPC v1.1.0)^98^.

In order to investigate the effect of c-tDCS on learning-induced transfer, pre-post difference scores were calculated for (1) the control tasks, (2) cognitive and (3) motor transfer tasks. Transfer task comparisons were conducted using a non-parametric Brunner-Munzel test (brunnermunzel package)^40^, whereas mixed ANOVAs (described above) were used for comparing control tasks accounting for multiple time points. All correlational analyses were performed using Spearman rank-based correlation. Type I error rate α was set at the conventional significance level of 0.05. Depending on the statistical test used, effect sizes are reported as Cohen’s *d* (small = 0.20, medium = 0.50, large = 0.80) or Cliff’s delta (δ)^99^ interpreted as small = 0.11; medium = 0.28; large = 0.43^100^.

### Conference presentation

The results of this study were previously presented at the 28th European Congress of Sports Science conference held on 4–7th July 2023, Paris, France. This manuscript has also been uploaded to a pre-print server (MS ID#: BIORXIV/2023/572904).

### Supplementary Information

Supplementary Information.

## Data Availability

Data will be made available upon requests addressed to corresponding authors (NP or MT).
