# Supplementary material for: Online stimulation of the prefrontal cortex during practice increases motor variability and modulates later cognitive transfer: a randomized, double-blinded and sham-controlled tDCS study
Source: Sci Rep. 2024 Aug 29;14:20162. doi: 10.1038/s41598-024-70857-x (PMC11364672; doi:10.1038/s41598-024-70857-x)
Supplement: Supplementary file 1 — Supplementary Information. [file 41598_2024_70857_MOESM1_ESM.doc]

**Title: Online stimulation of the prefrontal cortex during practice increases motor variability and modulates later cognitive transfer: a randomized, double-blinded and sham-controlled tDCS study (supplementary material)**

Nisha Maria Prabhu^*, a^, Nico Lehmann^a,b^, Elisabeth Kaminski^b,e^, Notger Müller^c,d,f^, Marco Taubert ^*,a,c^

^a^Faculty of Human sciences, Institute III, Department of Sport Science, Otto von Guericke University, Zschokkestraße 32, 39104 Magdeburg, Germany

^b^Department of Neurology, Max Planck Institute for Human Cognitive and Brain Sciences, Stephanstraße 1a, 04103 Leipzig, Germany

^c^Center for Behavioral and Brain Science (CBBS), Otto von Guericke University, Universitätsplatz 2, 39106 Magdeburg, Germany

^d^Research Group Degenerative and Chronic Diseases, Movement, Faculty of Health Sciences Brandenburg, University of Potsdam, Am Mühlenberg 9, 14476 Potsdam, Germany.

^e^Department of Movement Neuroscience, Faculty of Sport Science, Leipzig University, Leipzig, Germany

^f^ Neuroprotection Lab, German Center for Neurodegenerative Diseases (DZNE), Magdeburg, Germany; Center for Behavioral Brain Sciences, Magdeburg, Germany

Available ORCID IDs

Prabhu, Nisha Maria: 0000-0001-8391-5174

Lehmann, Nico: 0000-0002-3146-5084

Kaminski, Elisabeth: 0009-0007-8975-4552

Mueller, Notger: 0000-0002-5483-6423

Taubert, Marco: 0009-0001-8483-5894

*Corresponding authors email addresses: [nisha.prabhu@ovgu.de](mailto:nisha.prabhu@ovgu.de), [marco.taubert@ovgu.de](mailto:marco.taubert@ovgu.de)

**Supplementary material:**

1. **Methods**
   1. Sample size estimation: Sample size estimation for this study was based on the expected behavioural effect of tDCS on online DBT performance. In this respect, ^1^ revealed the sham group to significantly outperform the interfering stimulation group in terms of online learning performance with a large underlying effect size (*d* ≈ 0.9, as estimated from the reported F-statistic). Assuming a slightly lower effect size of *d* = 0.8, probability of type I error α = 0.05, and power = 0.8 yielded a sample size of n = 21 per group (two-sample t-test, one-tailed; ^2^).
   2. Control tasks:
      1. Balance Error Scoring System (BESS)^3^: This test includes maintaining various stances/positions executed bare feet, hands on the hips, eyes closed, initially on the floor followed by balancing on a medium density foam cushion. The different test positions comprised: double leg stance, single leg stance and a tandem stance. During testing, the participants were asked to maintain the test position for a duration of 20secs each with as few deviations as possible. Every deviation from the original position was marked as an error, with a maximum score of 60. As prescribed in the BESS protocol, errors included opening of eyes, stepping, moving out of position, hands leaving hips, etc. The summed-up errors from all the test positions in the pre and post-test at all three training sessions were used for analyses.
      2. Stroop test ^4,5^: To control for the effect of tDCS on general executive functions, a computerised version of Stroop test was administered using the Presentation® software by Neurobehavioral Systems, Inc., Berkeley, CA, USA. Coloured words reading red, blue, green or yellow were presented on a screen with a white background either in a congruent or incongruent font colour. Buttons on the keyboard pertaining to the respective colours were pre-assigned. The task included two conditions, viz., participants asked to respond by identifying either the colour of the word or the word itself by pressing the pre-assigned button as quickly as possible. Prior to testing, all participants went through familiarization trials. Accuracy as well as quick response were emphasized as good performance. The participants received immediate feedback after every trial as ‘correct’ or ‘incorrect’ response. A total of forty-eight trials per condition were presented in a randomized order (congruent/incongruent). For the purpose of analyses, an interference score was calculated (outcome measure: mean reaction time or accuracy for the incongruent condition subtracted from the congruent condition)
2. **Further analyses and results:**
   1. Stimulation questionnaire: Intensity of the sensations reported by subjects after tDCS (2.1.1 in the manuscript). Intensities for tingling, burning, headache, nausea and ill feeling were evaluated on a 5-point scale with 1: none at all, 2: mild, 3: moderate, 4: somewhat strong/ considerable, 5: very strong. The effect of tDCS on concentration, attention, alertness, mood change was rated on an inverted scale with 1: strong disturbance, 2: mild disturbance, 3: no effect, 4: mildly improved, 5: strongly improved (Supplementary Table S1).

Supplementary Table S1. Stimulation questionnaire responses for subjective perception of tDCS immediate effects. Values denote mean rating for each sensory perception separated for each group.

| **Training session** | **Condition** | **Tingling** | **Burning** | **Headache** | **Nausea** | **Feel ill** | **Concentration** | **Attention** | **Alertness** | **Mood change** |
| --- | --- | --- | --- | --- | --- | --- | --- | --- | --- | --- |
| **TD1** | s-tDCS | 2.22 | 1.61 | 1.52 | 1.05 | 1.24 | 1.52 | 3.01 | 3.08 | 3.05 |
| **TD1** | c-tDCS | 2.24 | 1.58 | 1.46 | 1.04 | 1.24 | 1.50 | 3.01 | 3.08 | 3.06 |
| **TD3** | s-tDCS | 2.25 | 1.62 | 1.52 | 1.05 | 1.25 | 1.54 | 3.00 | 3.07 | 3.05 |
| **TD3** | c-tDCS | 2.26 | 1.59 | 1.46 | 1.03 | 1.23 | 1.50 | 3.02 | 3.09 | 3.06 |
| **TD5** | s-tDCS | 2.27 | 1.62 | 1.52 | 1.05 | 1.25 | 1.52 | 3.01 | 3.06 | 3.04 |
| **TD5** | c-tDCS | 2.24 | 1.59 | 1.46 | 1.03 | 1.23 | 1.48 | 3.04 | 3.11 | 3.06 |

- 1. Blinding of stimulation: The blinding index (BI) on TD1 was estimated at 0.56 with 95% CI [0.42, 0.69], on TD3 BI = 0.44 with 95 % CI [0.32, 0.57] and on TD5 BI = 0.59 with 95% CI [0.49, 0.69], indicating random guessing. These results combined with the results of the stimulation questionnaire, indicate successful blinding between the groups (Supplementary figure S1).


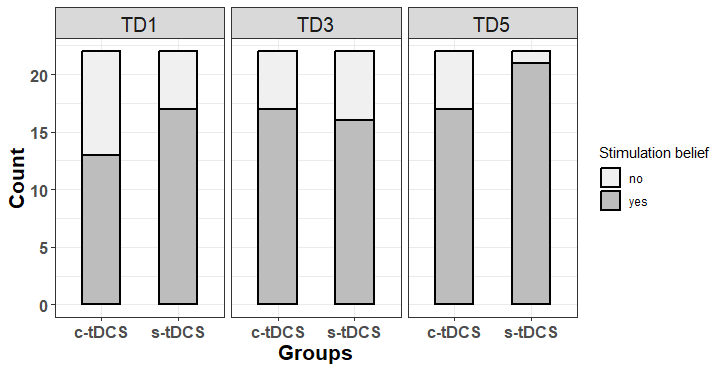


*Supplementary Figure S1. Responses from both groups about stimulation belief (blinding) on training sessions 1, 3, 5*

- 1. Stroop task: No difference was observed between groups in the accuracy interference part of the Stroop test, however in the reaction time part of this test, an effect of group without an interaction effect or time was observed (Supplementary figure S2 and 2.1.3 in manuscript).

(a)
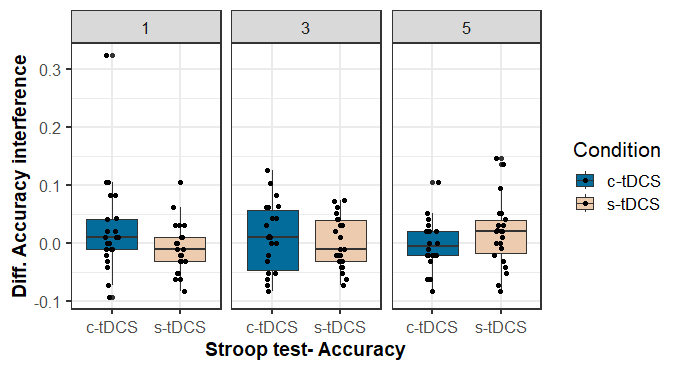
(b)
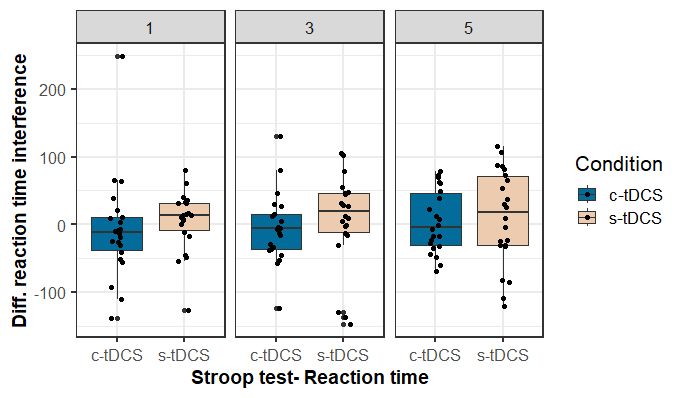


Supplementary Figure S2. Changes in general cognitive ability measured using the Stroop task. Y-axis shows pre-post difference scores calculated immediately before and after intervention on TD1, TD3, TD5 (a) accuracy interference= difference between incongruent and congruent responses; (b) reaction time interference= difference between reaction time of incongruent and congruent responses.

- 1. BESS score: No differences between groups were observed for this control measure of general balance ability (Supplementary figure S3 and 2.1.4 in manuscript).


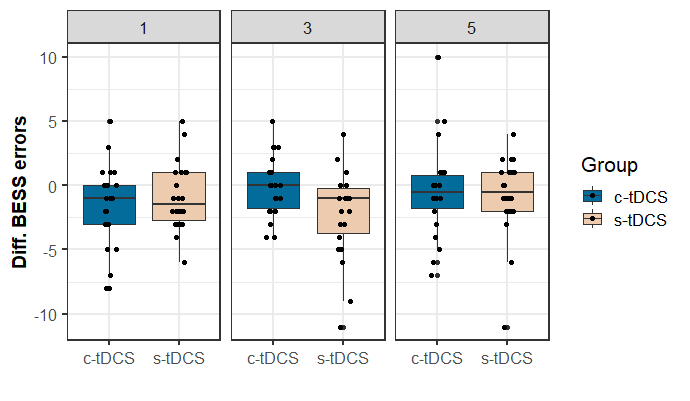


Supplementary Figure S3. Improvement in general balance ability measured using BESS. Y-axis shows pre-post difference scores calculated immediately before and after intervention on TD1, TD3, TD5.

- 1. Baseline DBT performance: Baseline performance recorded as the first 2 trials on TD1 (before tDCS stimulation commenced) was found to be similar between both groups (mean TIB c-tDCS: 3.05 ± 1.7 secs vs s-tDCS: 2.99 ± 1.49 secs), Brunner-Munzel *t*(41.97) = 0.27, *p* = .80, Cliff δ = 0.04 (Supplementary Figure S4a). The baseline performance variability (coefficient of variation - CoV) calculated from the first two trials on TD1 (before tDCS commenced) was similar between both groups (mean CoV c-tDCS: 0.30 ± 0.2 vs s-tDCS: 0.27 ± 0.14), Brunner-Munzel t(34.02) = -0.29, p = .77, δ = .05 (Supplementary Figure. S4b online).

1.
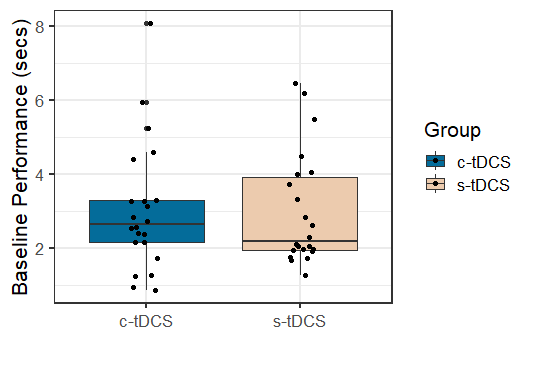
(b)
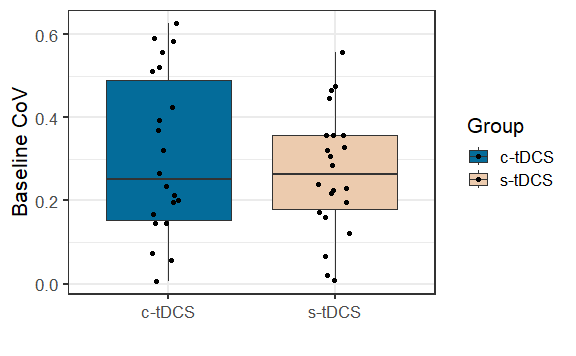


Supplementary Figure S4. (a) Baseline performance on the DBT recorded at the first 2 trials (without tDCS stimulation), was found to be similar between both groups. Y-axis represents the TIB in seconds. (b) Baseline CoV recorded at the first 2 trials (without tDCS stimulation), was similar between both groups.

- 1. Influence of initial performance on DBT improvements: Irrespective of group assignment, lower initial TIB performance led to higher performance improvements (*b* = -16.98, *t* = -3.41, *p* = .002) and increased performance variability (*b* = -0.01, *t* = -4.91, *p* < .0001). However, the effect of tDCS on performance variability remained significant when controlling for initial performance (*b* = -0.021, *t* = -2.48, *p* = .02). Whereas, there was no effect of tDCS on performance improvement (*b* = 10.59, *t* = 0.57, *p* = .57). Spearman rank based correlation analyses (Supplementary Figure S5) between baseline performance and performance improvement from the first to sixth training session showed a moderate negative correlation for both c-tDCS (*r* = -0.46, *p* = .03) and s-tDCS (*r* = -0.49, *p* = .02) groups. Furthermore, correlation between baseline performance and mean CoV over the entire training duration for c-tDCS group showed a moderate negative correlation (*r* = -0.52, *p* = .015), similarly the s-tDCS group showed a strong negative correlation of *r* = -0.67, *p* = .001. No significant differences were observed after comparing the correlation coefficients^6^ for both groups (performance change: *z* = 0.12, *p* = .9; mean CoV: *z* = 0.72, *p* = 0.47). Altogether, these results demonstrate that initial performance did not influence the observed between group differences after balance with/-out c-tDCS interventions.

(a)
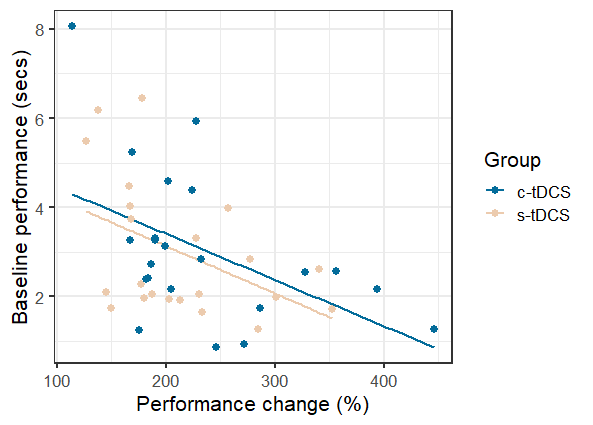
 (b)
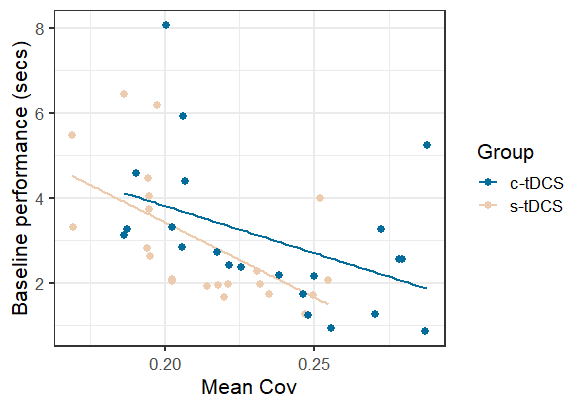


*Supplementary Figure S5. Correlation between baseline performance (TIB) on the stabilometer and (a) performance improvements (% change) from TD1 to TD6 expressed as percentage; (b) mean CoV over the entire training duration in the c-tDCS and s-tDCS group.*

- 1. Influence of baseline motor/balance skills on DBT performance: The Balance Error Scoring System (BESS) was used to test participants’ balance skills as it measures the ability of the individuals to minimize postural sway while systematically disrupting the sensory selection process by varying available somatosensory and/or visual information^3^. BESS pre-intervention scores were used to control for the baseline balance ability/skills. In this respect, multiple regression analyses with either mean performance improvements or mean performance variability as predicted variables and BESS values as well as tDCS as predictor variables revealed no significant influence of BESS on each predicted variable (performance improvement: *b* = 1.12, *t* = 0.33, *p* = .74; mean performance variability: *b* = 0.001, *t* = 1.23, *p* = .23). More importantly, the effect of tDCS on performance variability remained significant when controlling for BESS (*b* = 0.022, *t* = 2.14, *p* = .04). Whereas, there was still no significant effect of tDCS on performance improvement (*b* = 5.608, *t* = 0.23, *p* = .82) when controlling for BESS. Additionally, no between-groups differences were detected in their habitual physical activity levels (Table. 1 in the manuscript).
  2. Minimum values: A comparison of the minimum (lowest TIB) and maximum (highest TIB) values attained by every participant during the entire training duration revealed a progressive improvement in the minimum values over the training period *F*(5, 19.13) = 59.35, *p* = .00, *d*>1 , along with an effect of interaction *F*(5,19.13) = 3.45, *p* = .02, *d* = 0.57 without a group effect *F*(1, 25.49) = 0.42, *p* = .5 (Supplementary Figure S6). The maximum values did not exhibit any effects of interaction or group, instead only an effect of time was detected *F*(1, 19.11) = 18.86, *p* = .00, *d*>1.


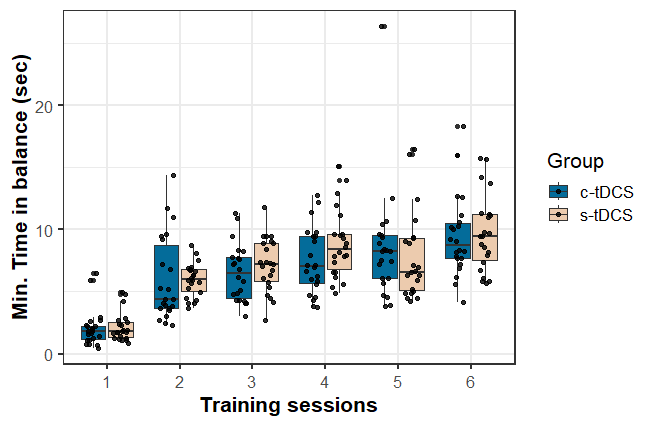


Supplementary Figure S6. Minimum TIB values exhibited by every participant in both groups over 6-training sessions.

- 1. Testing for potential ceiling effects: Due to the absence of a training effect on the maximum balance time, the possibility of ceiling effects was examined. However, at the 6^th^ training session it was observed that a total of three participants displayed an average TIB of over 20 secs from a possible 30 secs, i.e., two participants in the c-tDCS group recorded 23.8secs and 24.9secs, whereas only one participant from the s-tDCS group reached an average TIB of 21.8 secs. This along with findings shown in Supplementary Figure S7, suggests that although learning curves might have plateaued in some participants, there was still room for further improvements in all participants.

(a)
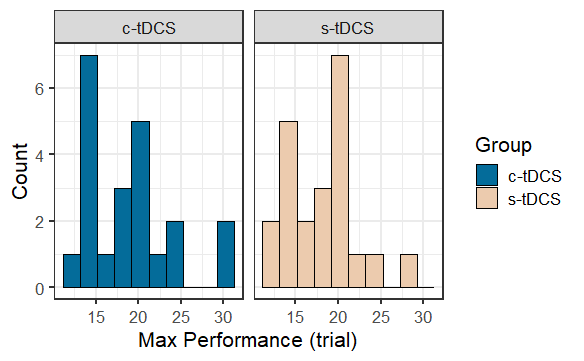
 (b)
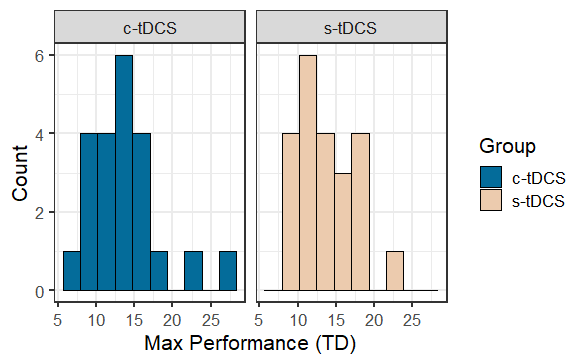


*Supplementary Figure S7. During the entire training duration, maximum time in balance (TIB) displayed (trial-wise in (a) and session-wise in (b)) for both groups. (a) The median of the maximum TIB recorded for c-tDCS group was 17.92 secs (IQR: 6.38) whereas s-tDCS recorded 18.63 secs (IQR: 6.34). (b) The median of the maximum TIB (averaging trials over each TD) for c-tDCS group was 13.31 secs (IQR: 5.51) whereas s-tDCS recorded 12.65 secs (IQR: 5.86).*

- 1. Cognitive and control tests:
     1. VVM: No differences between groups were found in this visuospatial memory test (Supplementary figure S8 and 2.3.1 in manuscript)

(a)
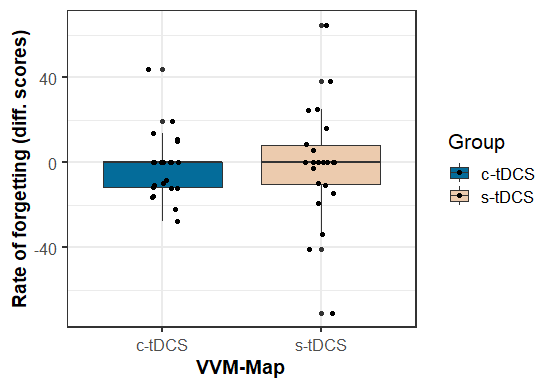
 (b)
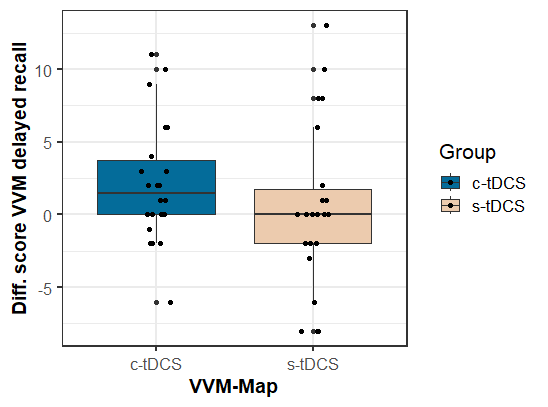


Supplementary Figure S8. Improvement in visuospatial memory task VVM seen as pre-post difference scores calculated from the pre and post test scores for (a) rate of forgetting and (b) delayed recall.

- - 1. Eriksen flanker task: A significant positive correlation was noticed between the accuracy interference score and the mean CoV only in the s-tDCS group (Spearman: *p* = .02, *r* = 0.48) implying participants with a lower variability experienced higher gains in accuracy by successfully reducing their accuracy interference (Supplementary Figure S9).


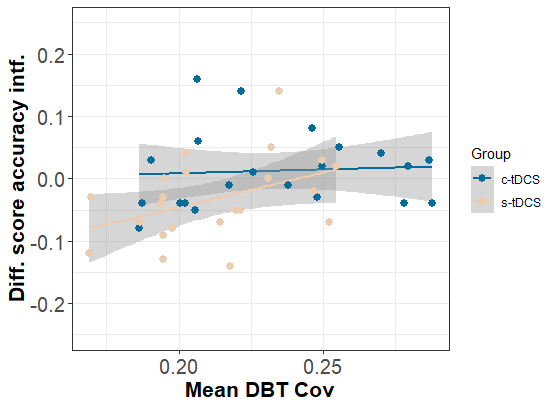


Supplementary Figure S9. Correlation between motor variability (CoV) on the stabilometer and the difference score in accuracy interference values in the c-tDCS and s-tDCS group. For purposes of better visualization an outlier with a score of (-0.68) from the c-tDCS group was removed from the graph.

**References:**

1. Kaminski, E. *et al.* Effect of transcranial direct current stimulation (tDCS) during complex whole body motor skill learning. *Neurosci. Lett.* **552**, 76–80 (2013).

2. Faul, F., Erdfelder, E., Lang, A. G. & Buchner, A. G*Power 3: A flexible statistical power analysis program for the social, behavioral, and biomedical sciences. *Behav. Res. Methods* **39**, 175–191 (2007).

3. Bell, D. R., Guskiewicz, K. M., Clark, M. A. & Padua, D. A. Systematic review of the balance error scoring system. *Sports Health* **3**, 287–295 (2011).

4. Golden, C. J. Stroop Effect. *Encycl. Clin. Neuropsychol.* 3327–3330 (2018) doi:10.1007/978-3-319-57111-9_1910/COVER.

5. Stroop, J. R. Studies of interference in serial verbal reactions. *J. Exp. Psychol.* **18**, 643–662 (1935).

6. Diedenhofen, B. & Musch, J. cocor: A Comprehensive Solution for the Statistical Comparison of Correlations. *PLoS One* **10**, e0121945 (2015).
